# Baseline IFN-γ and IL-10 expression in PBMCs could predict response to PD-1 checkpoint inhibitors in advanced melanoma patients

**DOI:** 10.1038/s41598-020-72711-2

**Published:** 2020-10-19

**Authors:** Emilio Francesco Giunta, Giusi Barra, Vincenzo De Falco, Giuseppe Argenziano, Stefania Napolitano, Pasquale Vitale, Nicoletta Zanaletti, Marinella Terminiello, Erika Martinelli, Floriana Morgillo, Davide Ciardiello, Raffaele De Palma, Fortunato Ciardiello, Teresa Troiani

**Affiliations:** 1 Department of Precision Medicine, University of Campania Luigi Vanvitelli, 80131 Naples, Italy; 2 Department of Mental and Physical Health and Preventive Medicine, University of Campania Luigi Vanvitelli, 80131 Naples, Italy; 3 Department of Internal Medicine, University of Genoa, IRCCS Ospedale Policlinico San Martino, 16132 Genoa, Italy

**Keywords:** Melanoma, Cancer immunotherapy, Tumour immunology, Cytokines, Tumour biomarkers, Cancer

## Abstract

Anti-PD-1 antibodies revolutionized the treatment of advanced melanoma patients. However, one out of three do not respond to this therapy, with an overall poor prognosis. Identification of predictive biomarkers in patients receiving immune-based therapies is necessary for minimizing risk of toxicity and optimizing patient benefit and is still an important unmet clinical need. Recently, many studies have evaluated peripheral blood markers as potential biomarkers, but none so far have been validated. We collected at baseline peripheral blood samples from 18 consecutive advanced melanoma patients treated with anti-PD-1 therapy. Main pro- and anti-inflammatory cytokines were studied in PBMCs from baseline blood samples both evaluating mRNA expression by qRT-PCR and identifying PBMCs subpopulations by FACS analysis. We found that IFN-γ mRNA expression levels were significantly higher in responder patients compared to non-responder ones. Moreover, to better validate its role, we evaluated the IFN-γ/IL-10 ratio. This value was higher in responder patients. FACS analysis confirmed that CD4 + IFN-γ + PBMCs percentage was higher in responders. Our data suggest an interesting correlation between IFN-γ/IL-10 ratio and response to anti-PD-1 therapy in advanced melanoma patients, suggesting a new biomarker that could be easily incorporated in clinical practice.

## Introduction

Malignant melanoma, even if accounting for only about 1% of skin cancer, is the most lethal type, with a 5-year relative survival rate of 23% for stage IV disease^1^. The introduction in clinical practice of immunotherapy has dramatically changed the outcome of patients with metastatic melanoma^2^. Ipilimumab, the first anti-CTLA-4 antibody used in metastatic melanoma, prolonged overall survival in pre-treated patients^3^, leading in August 2010, to its approval by FDA for this clinical setting. The treatment with ipilimumab has been subsequently replaced by anti-PD-1 antibodies, new immune checkpoint drugs that have showed a higher efficacy with lower toxicities. Based on phase III clinical trials, pembrolizumab and nivolumab, two anti-PD-1 antibodies, were approved by FDA in September and December 2014 respectively^4,5^. Despite efficacy of anti-PD-1 antibodies, a portion of patients treated with these drugs does not obtain any clinical benefit, showing instead rapid progressive disease and poor outcome. Specifically, 32.9% and 38% of the patients treated respectively with nivolumab and pembrolizumab achieved progressive disease as best overall response to treatment^4,5^. This means that at least one patient out of three does not benefit from anti-PD-1 therapy at all. Biological markers are parameters that could be objectively evaluated as indicators of pharmacologic responses to a therapeutic intervention^6^. Predictive biomarkers for response to anti-PD-1 antibodies nowadays still represent an unmet clinical need, since none of the investigated ones has demonstrated a clinically-useful role in distinguishing between responder and non-responder patients^7^. Moreover, the establishment of biomarkers for anti-PD-1 drugs could avoid potentially harmful drugs in patients who will not respond to them, aiding in the more precise delivery of immunotherapy.

Programmed death-ligand 1 (PD-L1) expression in melanoma tumor tissue has been investigated as a potential biomarker since this protein directly interacts with the target of anti-PD-1 therapy. However, several problems using PD-L1 expression could limit its use as a biomarker for immuno-therapy, such as heterogeneity within tumors, uncertainty about threshold levels and, most of all, the observation that some PD-L1 negative patients also have clinical benefit from treatment with an anti-PD-1/PD-L1 inhibitors^8^. In this scenario, many studies in the last years have explored emerging predictive biomarkers including the role of cytokines on both blood and tissue samples obtained from melanoma patients treated with immune checkpoint inhibitors^9^. In particular, the two most important cytokines that play a key role in immune-response regulation are IFN-γ and IL10. IFN-γ is a pro-inflammatory cytokine produced predominantly by T and NK cells, whereas IL10 is an anti-inflammatory one that can also be produced by tumor cells including melanoma^10,11^. The pro-inflammatory role of IFN-γ is mainly related to its ability to promote the T helper 1 (Th1), cytotoxic T lymphocytes (TCD8+) and NK activities. Moreover, it contributes to inhibit the expression of several anti-inflammatory cytokines including IL-10^12,13^. On the other hand, IL-10 is the cytokine mainly produced by T regulatory lymphocyte (Tregs) involved in the dampening of immune responses and the inhibition of the principal pro-inflammatory cytokines, including IFN-γ. So, IL-10 and IFN-γ are negatively correlated to each other^14^. However, even if the role of these cytokines in regulating the immune response is well clarified, their implication as potential biomarkers of response to immune check-point inhibitors is still controversial. In this scenario, we have investigated the role of baseline cytokine expression as predictive biomarkers of response to treatment with anti-PD-1 drugs in advanced melanoma patients.

## Methods

### Patients characteristics, clinical data and tumor assessment

**Figure 1:**
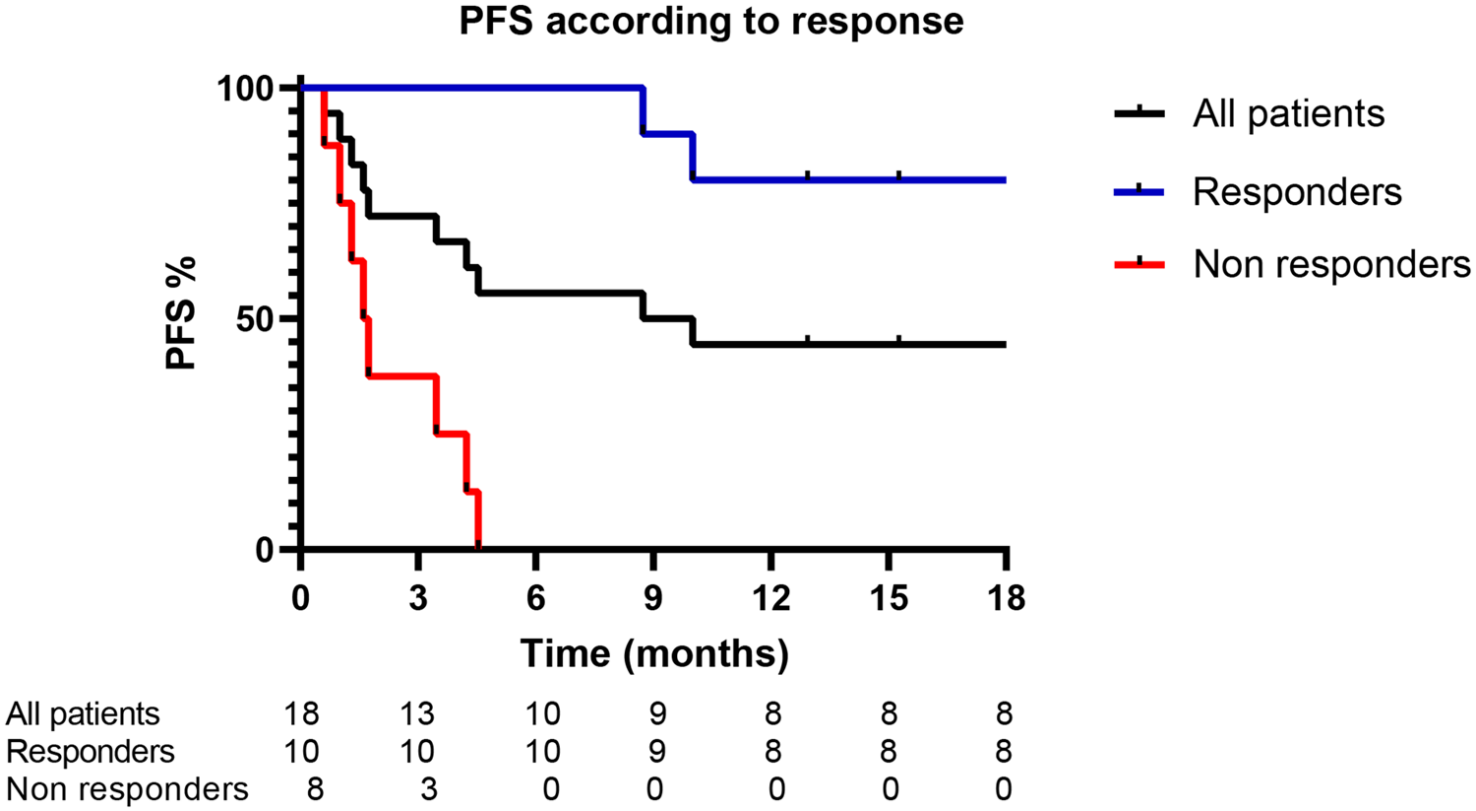
Kaplan–Meier curves for Progression Free Survival in the study population (black), responders (blue) and not responders (red).

Peripheral blood samples from 18 consecutive patients treated with anti-PD-1 therapy, 10 with nivolumab and 8 with pembrolizumab, for locally advanced or metastatic melanoma were collected in the Department of Oncology at University of Campania Luigi Vanvitelli between December 2016 and September 2018. Blood samples were collected at baseline, within 1 h before first infusion, after patients provided written informed consent. The study was approved by Ethics Committee of University of Campania Luigi Vanvitelli (Protocol n° 59) and conducted in accordance with the ethical principles of the Declaration of Helsinki. Patients received treatment until disease progression or intolerable toxicity. Tumor assessment was performed at baseline and every 12 weeks (± 4 weeks) and clinical response was classified according to immune-response evaluation criteria in solid tumors (iRECIST)^15^. The progression free survival (PFS) was defined as the interval between the start of anti-PD-1 therapy to clinical progression or death. Baseline demographical and clinical characteristics are listed in Table 1. Specifically, patients have been divided in two groups: 10 responder patients (R) (patients who achieved stable disease, SD, partial response, PR, or complete response, CR, as best response to treatment) and 8 non-responder patients (NR) (patients who achieved progressive disease, PD, as best response to treatment, excluding pseudo-progression). The median age of patients enrolled was similar between the two groups (median: 68 vs 61.5 years), with a predominance of females in the R group (66.6%) and males in the NR group (75%). Regarding stage of disease (according to AJCC staging system, VIII edition), in NR group 3 out of 10 patients, (37,5%) had M1d disease, contrary to R group, where 2 out of 10 patients (20%) had M1b as maximal tumor. Based on tumor molecular profile, responder patients were all BRAF wild type, whereas non-responder patients, harbored BRAF V600 mutation in 4 cases (50%). All BRAF V600 mutant melanoma patients received BRAF and MEK inhibitors combined treatment before starting anti-PD-1 based therapy (Table 1). In R group two patients had CR (20%), six PR (60%) and two SD (20%) with a disease control rate achieved in all patients. However, in NR group all patients achieved a PD as best responses to anti-PD-1 drugs. At September 2019, the data cut-off date, two patients died in the responders, one for disease progression (11 months after starting anti-PD-1 treatment) and the other for cardiovascular disease (8.7 months after starting treatment). On the contrary, 6 patients in non-responders group died for disease progression. According to efficacy data, the median PFS was not been reached (range: 8.7–not reached) in R group, whereas in NR group, median PFS was 1.7 months (range 0.6–4.5 months) (Table 1, Fig. 1).

**Table 1:** Baseline advanced melanoma patient characteristics in NR and R groups. (loc adv: locally advanced; PFS: progression free survival).

	Non-responders (NR, 8)	Responders (R, 10)
Age years: median (range)	61.5 (42–82)	68 (41–82)
Sex (M:F)	2:3	3:1
M0 (loc adv)	1 (12.5%)	2 (20%)
M1a	2 (25%)	6 (60%)
M1b	0 (0%)	2 (20%)
M1c	2 (25%)	0 (0%)
M1d	3 (37.5%)	0 (0%)
BRAF V600 mut	4 (50%)	0 (0%)
NRAS mut	1 (12.5%)	2 (20%)
Previous BRAFi + MEKi	3 (37.5%)	0 (0%)
PFS months: median (range)	1.7 (0.6–4.5)	not reached (8.7–not reached)

### PBMCS isolation, mRNA and FACS analysis

Peripheral blood mononuclear cells (PBMCs) were isolated from peripheral blood of patients by density gradient separation through Ficoll-Paque Plus (GE Healthcare), according to manufacturer instructions. Samples were collected before the first dose of anti-PD-1. Total RNA was extracted from PBMCs using Trizol reagent (Thermo Fisher). Reverse transcriptase reaction was carried out to convert 1 μg of isolated RNA into cDNA using SensiFAST cDNA synthesis (Bioline) according to the manufacturer instruction. Expression levels of genes encoding for IFN-γ, IL-10, IL-12, TNF-α and IL-4 were analysed using quantitative Real Time PCR (qRT-PCR). Gene-specific primers were designed by using PRIMER EXPRESS software (Applied Biosystems). Amplifications were done using the SensiFAST SYBR green (Bioline). The thermal cycling conditions were composed of 95 °C for 2 min followed by 40 cycles at 95 °C for 5 s and 60 °C for 20 s. All samples were run in duplicate, in 20 μL reactions using a Quant studio 7 flex (Applied Biosystems). 18S gene was used as internal control. To calculate relative gene expression in value it was used the 2−ΔCt method. Nonspecific signals caused by primer dimers were excluded by dissociation curve analysis and use of non-template controls.

PBMCs were cultured in in complete medium composed by RPMI 1640 containing human AB serum (10%), glutamine I (1%), penicillin and streptomycin (1%) for 6 h in the presence of phorbol 12-myristate 13-acetate (PMA, 10 ng/mL), Ionomycin (500 ng/mL) and Brefeldin A (BFA 10 μg/mL) (Sigma Aldrich). After this incubation, cells were washed in staining buffer (SB) (2% FBS; 0.1% sodium azide in PBS) and stained for 30 min with monoclonal antibody anti-CD45 Fitc (BD bioscience—Cod: 347463; Clone: 2D1), anti-CD3 Percp (BD Bioscience—Cod: 345766; Clone: SK7), anti-CD8 APC R700 (BD Bioscience—Cod: 565166; Clone: RPA-T8) and anti-CD4 PeCy7 (BD bioscience—Cod: 557852; Clone: SK3).

Cells were washed 2 times in SB and then fixed and permeabilized using BD cytofix/cytoperm to allow the intracellular staining of cytokines. PBMCs were stained again for 30 min with monoclonal antibodies anti-IL-10 Pe (BD Bioscience—Cod: 554706; Clone: JES3-19F1) and anti-IFN-γ Fitc (Miltenyi biotech—130-113-492; Clone: 45-15) and after 2 washes in 1X perm/wash, were acquired at Fortessa X20 flow cytometer (Becton Dickinson) and analysed with FACS DIVA software. Examples of gating strategy are reported in supplementary files.

Moreover, absolute lymphocyte count (ALC) and absolute neutrophil count (ANC) for each patient were collected before starting anti-PD-1. Baseline NLR (neutrophil to lymphocyte ratio) has been calculated as ANC/ANL ratio.

### Statistical analysis

The comparisons of cytokines levels, % of PBMCs, NLR and ratios between R and NR groups were analysed using unpaired two tail T test. The non-parametric unpaired two tail T test was chosen after checking normal distributions in each group, since dependent variables in the two independent groups were measured on an incremental level, assuming the null hypothesis (H0) of no significant difference between the means of the two groups.

The Receiver Operating Characteristic (ROC) curve was used to establish the cut-off values for mRNA and FACS analysis results according to the response to therapy (R/NR), assuming the null hypothesis (H0) of AUC = 0.5. The survival rate was estimated by Kaplan–Meier method, and the significance of the split between survival curves were measured by the log-rank (Mantel-Cox) test. P < 0.05 was considered to indicate a statistically significant difference. All statistical analyses were performed with GraphPad Prism 8.0.1 software.

## Results

### IFN and IL-10 mRNA expression predict response to anti-PD-1 therapy

**Figure 2:**
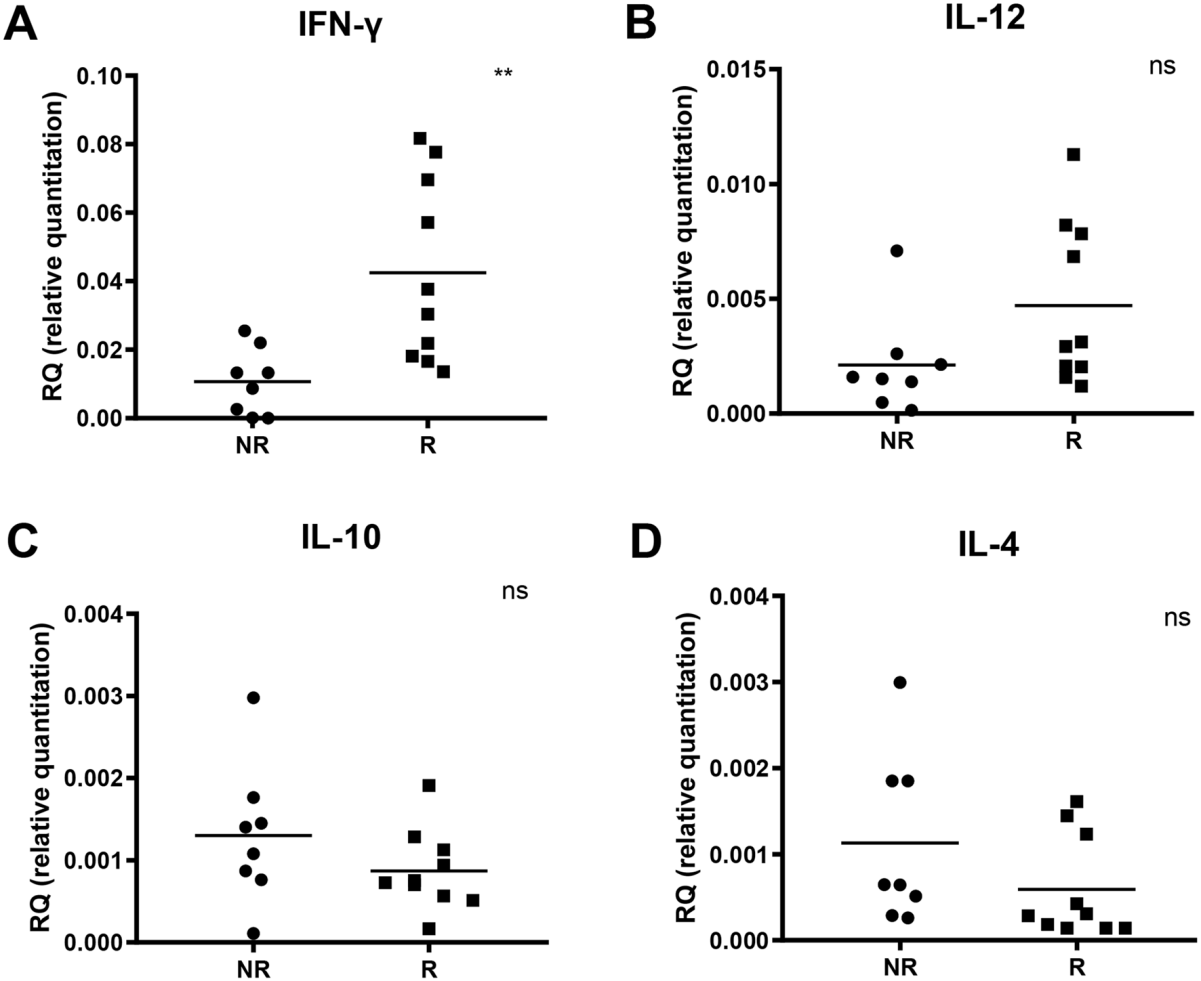
Baseline mRNA levels (qRT-PCR analysis) for each cytokine and their differences between NR and R groups. Baseline mRNA levels of IFN-γ (**A**), IL-12 (**B**), IL-10 (**C**) and IL-4 (**D**), respectively. Ns indicates not statistically significant, p > 0.05; ** indicates p < 0.01.

**Figure 3:**
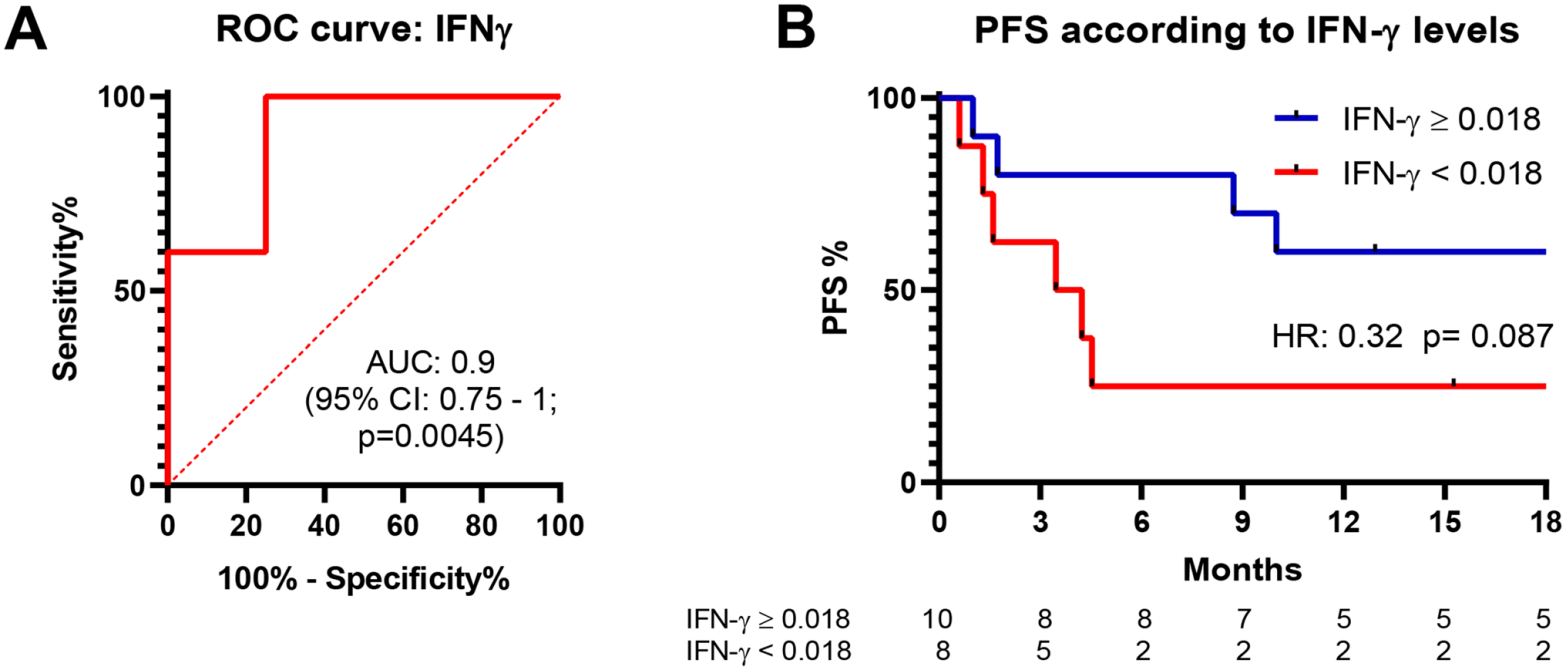
Analysis of ROC curve and PFS of patients according to their IFN-γ mRNA levels. ROC curve related to IFN-γ mRNA levels (null hypothesis for ROC analysis: AUC = 0.5) (**A**) and PFS analysis based on IFN-γ mRNA level expression (**B**).

To identify potential biomarkers that could be useful to predict response to anti-PD-1 drugs, we examined mRNA expression levels of a panel of pro- and anti-inflammatory cytokines in PBMCs from melanoma patients before first dose of anti-PD-1 treatment and compared these levels between R and NR groups. We found that levels of IL-10, IL-12 and IL-4 were similar between the two groups (p > 0.05), whilst levels of IFN-γ were significantly higher in R group compared to NR group (Fig. 2). In particular, IFN-γ mRNA levels showed a greater expression in R group (median: 0.034; range 0.013–0.081) than in NR group (median: 0.011; range: 0.0001–0.025, p = 0.006). ROC curve with IFN-γ mRNA levels, using a threshold of 0.018, showed positive predictive value (PPV) of 80% and negative predictive value (NPV) of 75% (Fig. 3A). Moreover, the AUC was 0.9 (95% CI: 0.75–1, p = 0,0,045) and HR for PFS was 0.32 (95% CI: 0.08–1.18, p = 0.087) (Fig. 3B).

**Figure 4:**
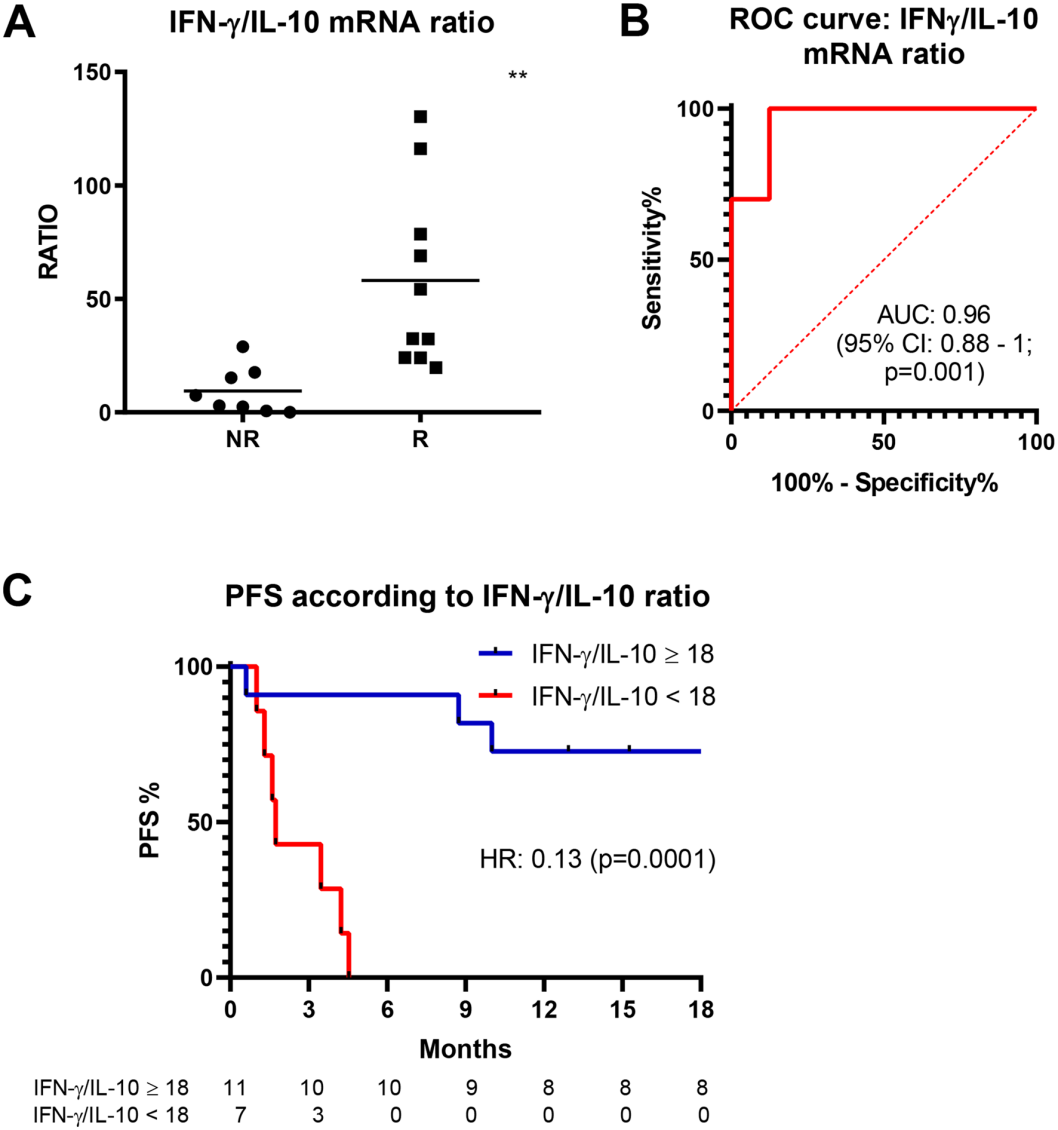
Baseline IFN-γ/IL-10 mRNA ratio and its predictiveness of response. IFN-γ/IL-10 ratio value in NR and R groups (**A**) and ROC curve analysis (null hypothesis for ROC analysis: AUC = 0.5) (**B**). PFS analysis using IFN-γ/IL-10 mRNA ratio value of 18 as threshold (**C**). ** indicates p < 0.01.

Based on these results, we combined data from single patients in order to increase PPV and NPV of IFN-γ mRNA levels. The ratio between IFN-γ and each single interleukin was evaluated (data not shown). Interestingly, only IFN-γ/IL-10 ratio value was statistically significant different between R and NR, with greater value in R group (median: 43.35; range: 19.72–130.33) than in NR group (median: 5.22; range: 0.07–28.91), p = 0.004 (Fig. 4A). As depicted in Fig. 4B, ROC curve related to IFN-γ/IL-10 ratio was calculated. Using a threshold of 18, PPV was 91% and NPV was 100%, AUC was 0.963 (95% CI 0.878–1, p = 0.001) (Fig. 4B). In addition, HR for PFS, using the same threshold as cut-off value, was 0.13 (95% CI: 0.03–0.56, p = 0.0001) (Fig. 4C).

### PBMCs subpopulations analysis predict response to anti-PD-1 therapy

Given the differences observed in IFN-γ and IL-10 mRNA levels between responders and non-responders, we analysed different subpopulations of PBMCs responsible for their production and secretion.

**Figure 5:**
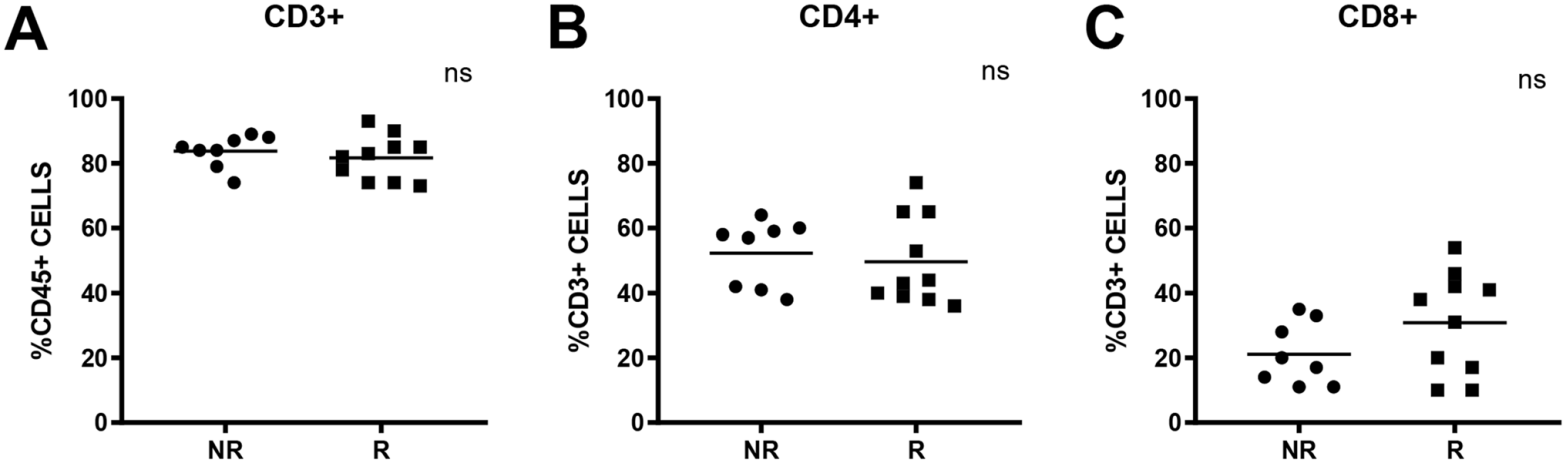
Baseline PBMCs subpopulations percentages and their differences between NR and R groups. CD3 + PBMCs subpopulation (**A**), CD3 + CD4 + PBMCs subpopulation (**B**) and CD3 + CD8 + PBMCs (**C**). N.s: not statistically significant.

**Figure 6:**
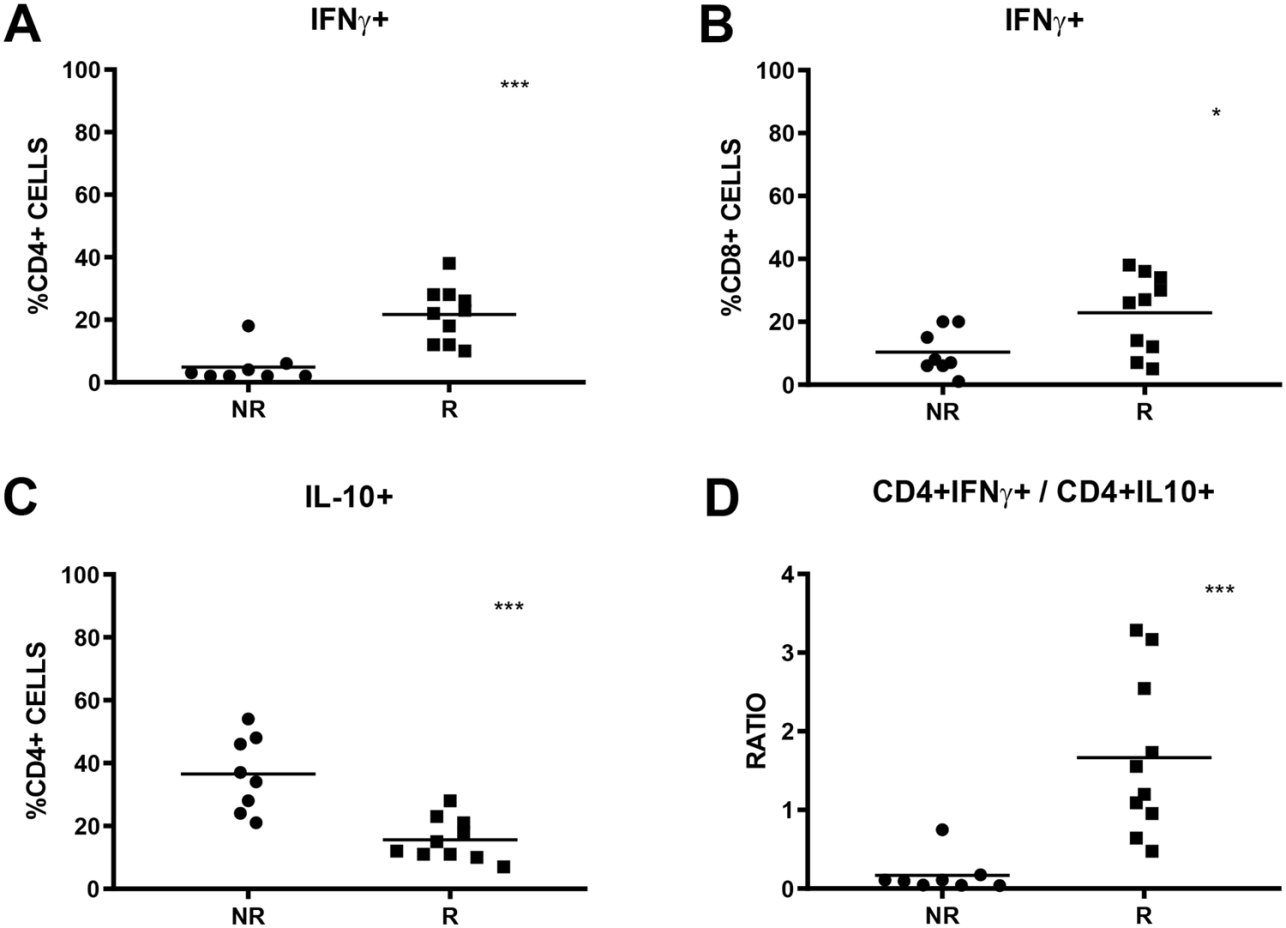
IFN-γ + and IL-10 + PBMCs subpopulations and CD4 + IFN-γ/CD4 + IL-10 ratio. Percentages of baseline CD4 + IFN-γ + PBMCs (**A**), percentages of baseline CD8 + IFN-γ + PBMCs (**B**), percentage of baseline CD4 + IL-10 + PBMCs (**C**). Baseline CD4 + IFN-γ/CD4 + IL-10 ratio (**D**). * indicates p < 0.05; *** indicates p < 0.001.

First, we assessed the amount of CD3 + T lymphocytes in the baseline samples. No significant differences were observed between the two groups of patients (Fig. 5A). Subsequently, we analysed the percentage of T cells positive for CD4 and CD8, markers associated with the two main lymphocytes subpopulations, T helper and T cytotoxic, respectively. Again, no significant differences were observed between the two groups of patients (Fig. 5B,C). Furtherly, we evaluated baseline IFN-γ + PBMCs in CD4 and CD8 positive lymphocytes. Notably, even if the percentage of CD4 + IFN-γ + and CD8 + IFN-γ + PBMCs subpopulations was statistically significant between R and NR groups, a better result was found for CD4 + IFN-γ + one. In fact, median percentage for R group was 22.5% (range: 12–38%) and 2.5% for NR group (range: 2–18%) respectively, p = 0.0002  (Fig. 6A,B). Based on these results, we also evaluated the expression of IL-10 in both CD4 and CD8 positive lymphocytes subpopulation. FACS analysis revealed a statistically significant difference in percentages only for CD4 + IL-10 + subpopulation. In particular, higher values were observed in NR group (median: 35.5%; range: 21–54%) than in R group (median: 13.5%; range: 7–28%), p = 0.0002  (Fig. 6C).

To evaluate the role of IFN-γ/IL-10 ratio to predict response of anti-PD-1 therapy, we combined data for CD4 + IFN-γ + and CD4 + IL-10 + as we previously demonstrated for cytokine mRNA ex-pression. CD4 + IFN-γ/CD4 + IL-10 ratio value was higher in R group (median: 1.38; range: 0.48–3.29) compared to NR group (median: 0.1; range: 0.04–0.75), p = 0.0009  (Fig. 6D).

**Figure 7:**
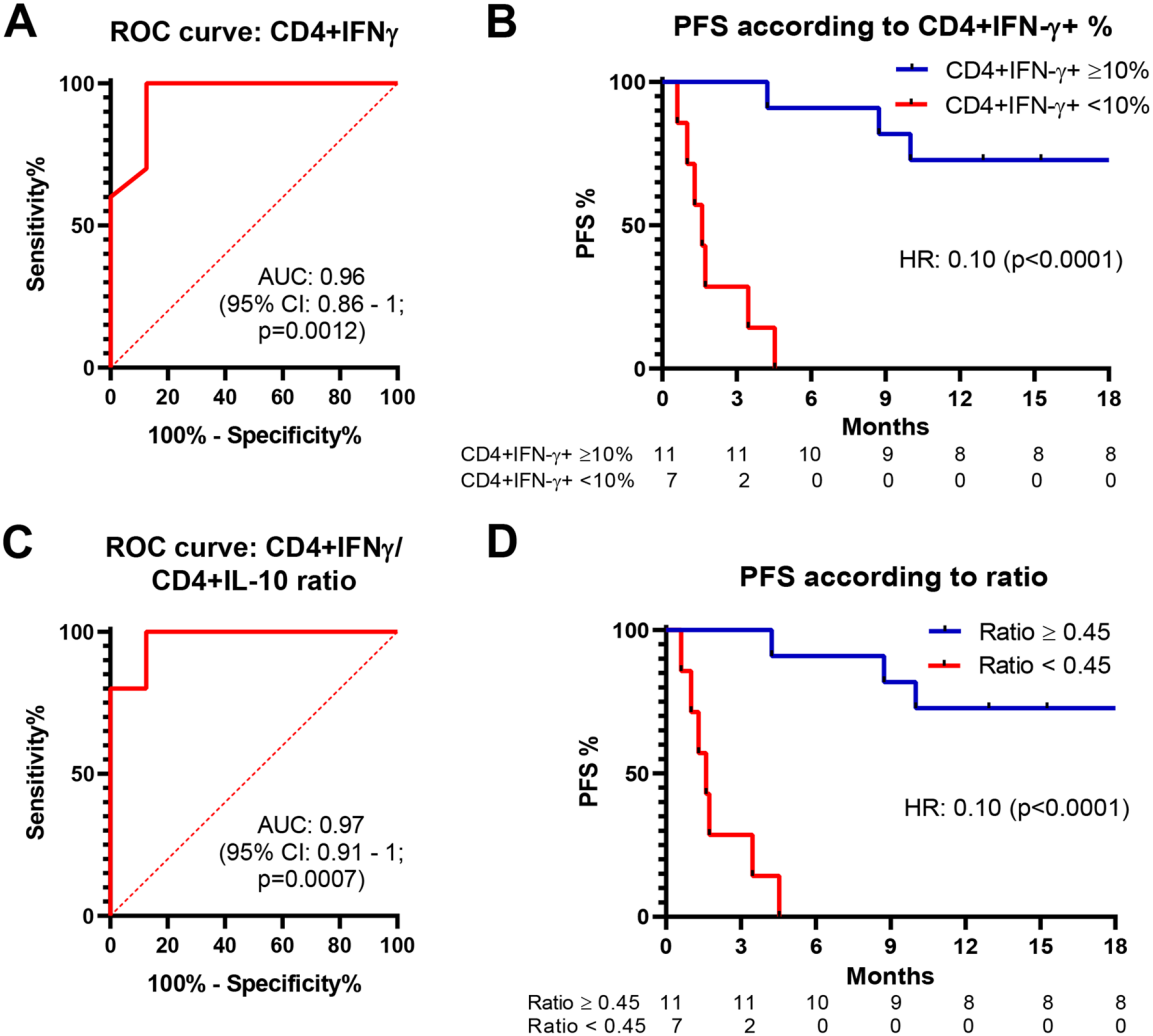
Predictiveness of response of baseline CD4 + IFN-γ subpopulation and CD4 + IFN-γ/CD4 + IL-10 ratio. ROC curve (null hypothesis for ROC analysis: AUC = 0.5) and PFS analysis of patients according to baseline CD4 + IFN-γ + PBMCs (**A**,**B**). ROC curve (null hypothesis for ROC analysis: AUC = 0.5) and PFS analysis of patients according to baseline CD4 + IFN-γ + /CD4 + IL-10 + ratio (**C**,**D**).

Finally, considering the two most statistically significative groups (CD4 + IFN-γ + and CD4 + IFN-γ/CD4 + IL-10), we performed ROC curves and PFS analysis on them. PPV and NPV were similar in both groups (91% and 100%, respectively), as well as HR value for PFS (0.10, 95% CI 0.02–0.48, p < 0.0001) (Fig. 7).

### Neutrophil to lymphocyte ratio seems to be independent from cytokines levels and ratios

**Figure 8:**
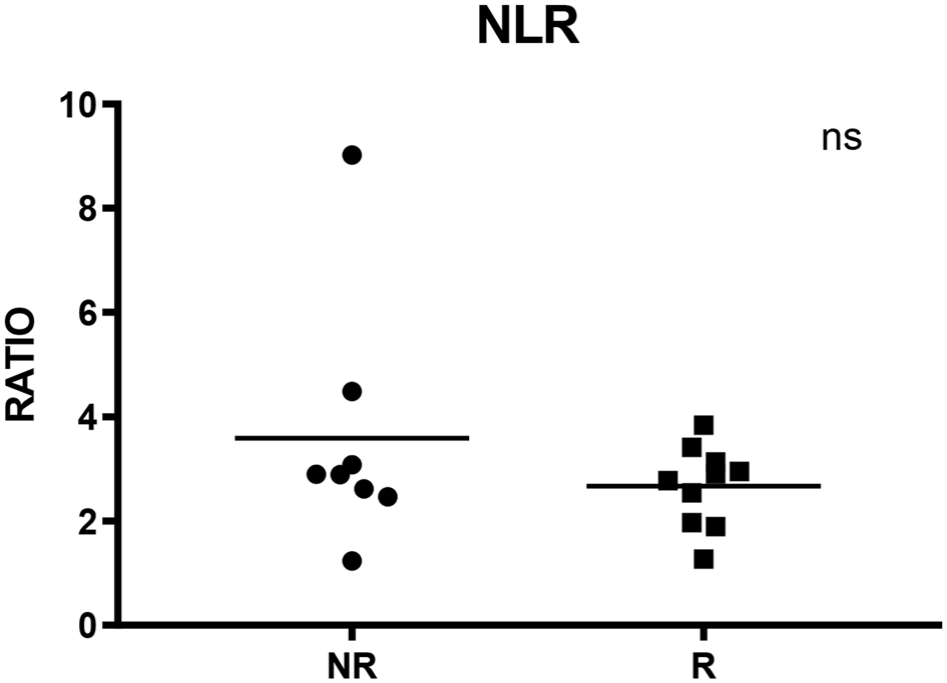
Neutrophil to Lymphocyte ratio in NR and R groups. Ns indicates not statistically significant.

Neutrophil to lymphocyte ratio (NLR), which is used as a marker of subclinical inflammation, has been evaluated in our cohort of patients since it was found to be an independent prognostic factor for adverse outcomes in solid tumours including melanoma. However, in our cohort of patients, distribution of baseline NLR showed no statistically significant difference among NR group (median: 2.89; range: 1.24–9.03) and R group (median: 2.84; range: 1.27–3.84), p = 0.26 (Fig. 8).

## Discussion

Cytokines are protein involved in immune and inflammatory responses in physiologic and pathologic conditions^16^. In the complex interaction between immune system and cancer, cytokines may play a primary role either in the tumor microenvironment, by directly activating/inactivating immune cells, and in the whole organism, by stimulating/inhibiting inflammation and other metabolic processes^17^.

IFN-γ and IL-10 are two cytokines involved in the regulation of T cells activity, playing an opposite role, so we focused on their expression on PBMCs to evaluate a correlation with tumor response in advanced melanoma patients treated with anti-PD-1 drugs.

IFN-γ plays a key role in antitumor activity through several mechanisms. In particular, it enhances Th1 differentiation and cytotoxic T lymphocyte (CTL) function and also inhibits immune regulatory activity of CD4 + CD25 + T cells (Tregs)^18^. Moreover, IFN-γ regulates tumor microenvironment, inhibiting suppressive functions of MDSC and downregulating angiogenesis processes^19–21^.

High baseline expression of IFN-γ in PBMCs, in advanced melanoma patients responding to anti-PD-1 therapy may indicate that the immune system of these patients is already set to attack cancer cells (inflammatory or anti-tumoral mood) and, probably, the concomitant inhibition of anti-PD-1 is the “spark that lights the fire”, removing the brake that inhibits effector T cells against tumor.

However, IL-10, differently from IFN-γ, has a controversial role in tumor-immune regulation^22^. In fact, it is produced and released in the tumor microenvironment by TAMs, dendritic cells (DCs), lymphocytes (especially Tregs) and/or tumor cells and promotes tumor immune escape mechanisms by down-regulation of MHC class II expression with consequent antigen presentation reduction^23,24^. On the other hand, it activates NK cells and increase cytotoxic functions^25,26^.

However, despite conflicting evidences, high levels of serum IL-10 have been associated with poor prognosis in cancer patients across different tumor types including melanoma^27–29^.

Our findings support the anti-inflammatory role of IL-10. In fact, high levels of this cytokine could probably reflect an anergic state of immune system (immune ignorance/tolerance), independently from number and sites of metastasis. A previous work showed that IL-10 receptor (IL-10R) on CD8 + T cells is upregulated using anti-PD-1 drugs in melanoma patients^30^. This could lead to immune suppression effect in metastatic melanoma patients with high IL-10 expression in PBMCs by using the PD-1 blockade agents. In order to study new biomarkers that could be used as predictive correlate for anti-tumor activity of nivolumab in immunotherapy-naïve metastatic melanoma patients, a phase 2 clinical trial has been carried out. In this trial authors examined cytokine level before nivolumab treatment. IFN-γ, IL6 and IL10 were significantly higher in responder patients^31^. However, IL-10 levels after 43 days from first dose of nivolumab did not show statistically significant variation, neither in responder or in non-responder patients. The discordance between our results and these ones could be explained by the use of different approaches. In this clinical trial authors evaluated serum cytokines: this method could probably be less “specific” than our analysis based on mRNA expression measurement in PBMCs. In fact, serum levels are highly variable due to their short half-life compared to longer half-life of mononucleate cells^32^.

Finally, in this work, to identify new biomarkers that could be useful to predict response to anti-PD-1 drugs, we examined mRNA expression levels of a panel of pro- and anti-inflammatory cytokines in PBMCs from melanoma patients before first dose of nivolumab treatment, including IL-10, IL-12 and IL-4 and IFN-γ. Levels of IFN-γ were significantly higher in R group compared to NR group, whilst IL-10, IL-12 and IL-4 levels were similar between R groups and NR groups (p > 0.05). In addition, we evaluated the ratio between IFN-γ and each single interleukin. Interestingly, only IFN-γ/IL-10 ratio value was statistically significant different between R and NR. Subsequently, we analysed different subpopulations of PBMCs responsible for cytokine production and secretion. We found that percentage of CD4 + IFN-γ + PBMCs are higher in R group and percentages of CD4 + IL-10 + PBMCs are more represented in NR group.

IFN-γ/IL-10 ratio has been described in several works focusing on infectious disease, such as TBC and HIV^33–36^, but also on autoimmune disease and transplants^37^.

**Figure 9:**
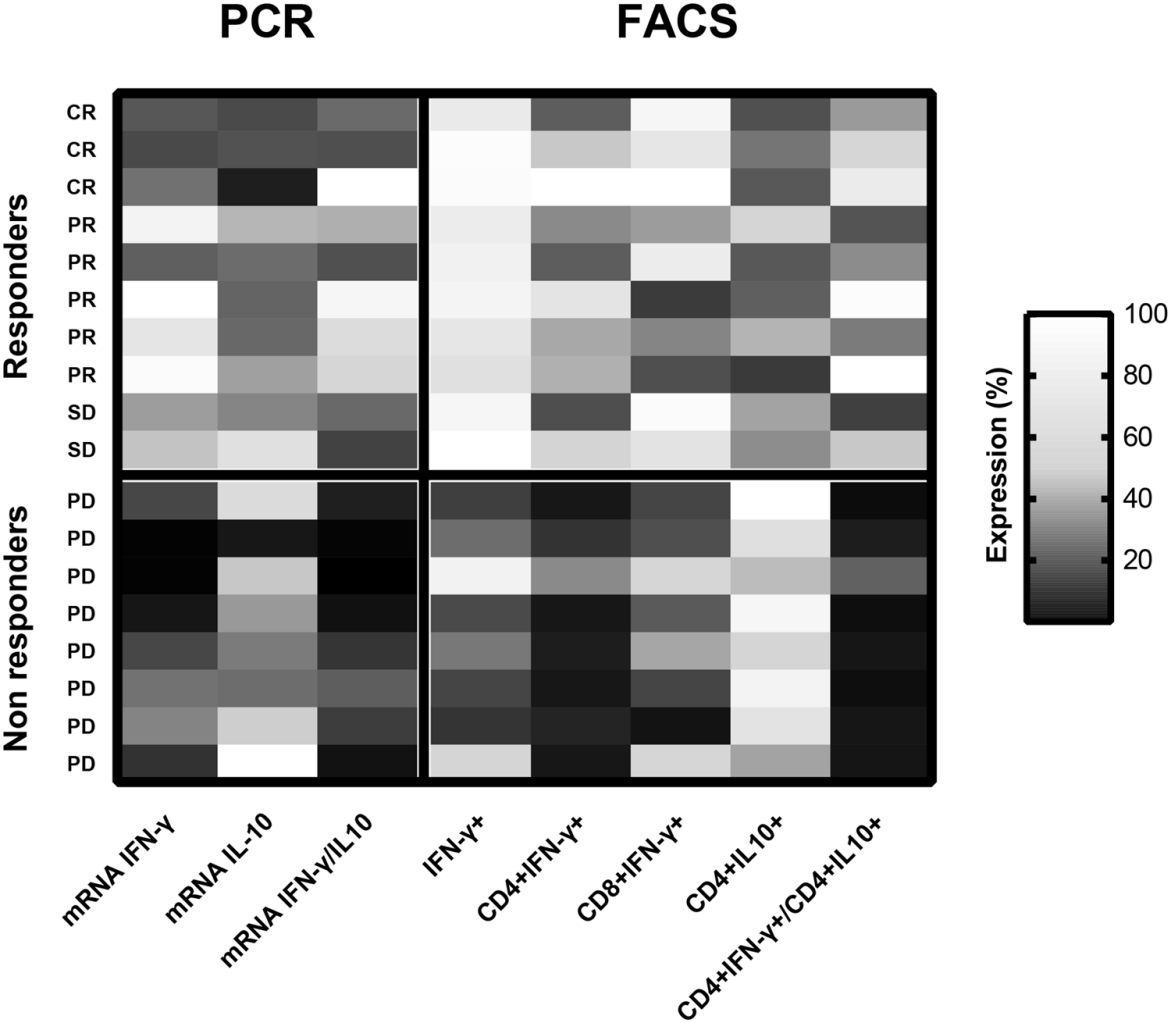
Heatmap showing results of qRT-PCR and FACS analysis.

**Figure 10:**
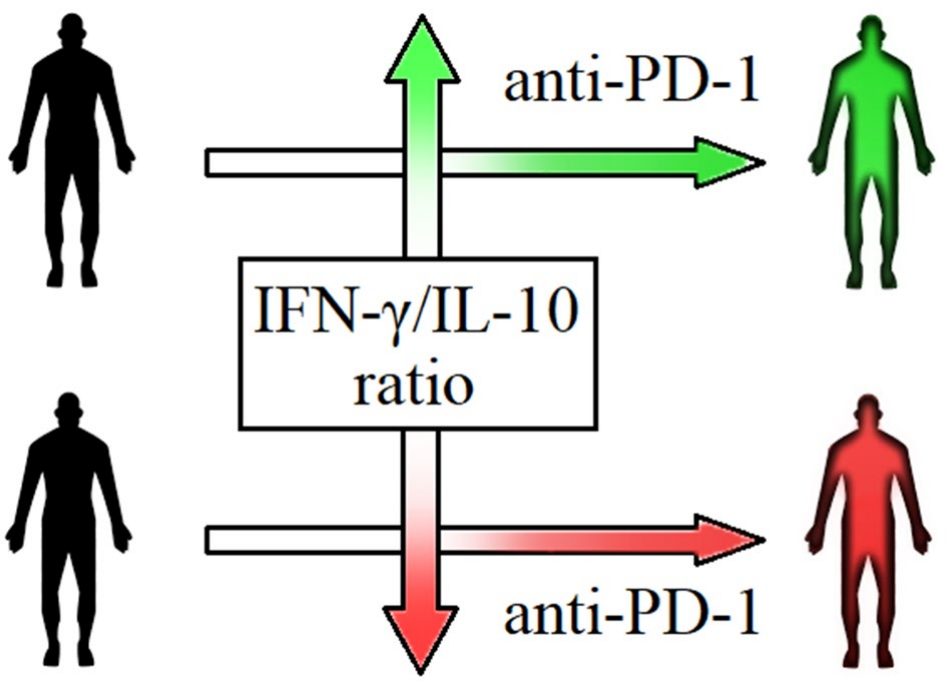
Predictive role of baseline IFN-γ/IL-10 ratio in advanced melanoma patients treated with anti-PD-1 therapy.

On the basis that, in our cohort of patients, mRNA IFN-γ/IL-10 ratio showed a better prediction of response to anti-PD-1 than IFN-γ mRNA levels alone, we evaluated CD4 + IFN-γ + /CD4 + IL-10 + ratio as biomarker to predict response to anti-PD-1 therapy. Basal CD4 + IFN-γ + /CD4 + IL-10 + ratio has the same predictive value (ROC AUC = 0.975) of basal mRNA IFN-γ/IL-10 ratio (ROC AUC = 0.9625). IFN-γ/IL-10 ratio, both on mRNA values or PBMCs percentages, could therefore represent a useful predictive tool since it balances, in the same patient, pro- and anti-inflammatory cytokines, showing a more realistic “photography” of immune system compared to single cytokines alone (Figs. 9 and 10). Patients with low IFN-γ/IL-10 ratio have lower probability of response to anti-PD-1 therapy. This could be explained by their impaired cytolytic T-cell activity, the last step of the “immunity cycle”^38^. However, they may respond to other immunotherapeutic agents, such as anti-CTLA-4 drugs, since different mechanisms, in different steps, are involved in the generation of anticancer immunity. Specifically, in our cohort of patients, 2 out of 8 NR patients received anti-CTLA-4 treatment after anti-PD-1 failure, with good clinical outcomes: at data cut off, SD and PR were the best responses obtained. These observations could probably suggest that PD-1/PD-L1 axis is not the only immune-evasion mechanism in these patients and that a low IFN-γ/IL-10 ratio could not exclude efficacy of other types of immunotherapy. In support of this argument, a previous work demonstrated that the inhibition of CTLA-4 could restore efficient T-cell priming by mature DCs after that their antigen presentation processes was inhibited by IL-10^39^.

Baseline neutrophil-to-lymphocyte ratio (NLR) has been investigated in several advanced solid tumors, including melanoma, as predictive biomarker for response to therapy. A recent meta-analysis of 12 studies in melanoma patients demonstrated that a high NLR was predictive of poor OS and PFS^40^. Moreover, NLR has been associated with OS in a retrospective analysis of 97 patients with stage IV melanoma treated with nivolumab in a single Italian institution^41^. In our cohort of patients, no statistically significant difference in median NLR was observed between R and NR groups, suggesting that variations in relative and absolute count of these white blood cells does not apparently affect percentages of PBMCs expressing IFN-γ and IL-10.

Limitations of this study are the retrospective nature of this analysis, the low number of patients included (18), the imbalanced distribution of M1 categories and BRAF-V600 mutant percentages among subgroups, the lack of adjustment for potentially confounding factors.

To our knowledge this is the first work that has demonstrated the potential use of a cytokine ratio as a new predictive biomarker of responsiveness to anti-PD-1 therapy in advanced melanoma patients. Both IFN-γ/IL-10 mRNA ratio and CD4 + subpopulations ratio in PBMCs could predict response to anti-PD-1 in immunotherapy-naive melanoma patients. These results must be taken with caution since this is a retrospective analysis with either a small sample size and an imbalanced distribution between the two groups of patients. However, the techniques and technologies used for our analysis are widespread and feasible for many laboratories indicating routinely clinical feasibility of these procedures. A future prospective validation of IFN-γ/IL-10 ratio in a larger population of advanced melanoma patients must be performed in order to better select patients who are candidate for anti-PD-1 therapy.

## Supplementary information

Supplementary file1

Supplementary file2

Supplementary file3
