# Supplementary figures and images for: Baseline IFN-γ and IL-10 expression in PBMCs could predict response to PD-1 checkpoint inhibitors in advanced melanoma patients
Source: Sci Rep. 2020 Oct 19;10:17626. doi: 10.1038/s41598-020-72711-2 (PMC7573589; doi:10.1038/s41598-020-72711-2)

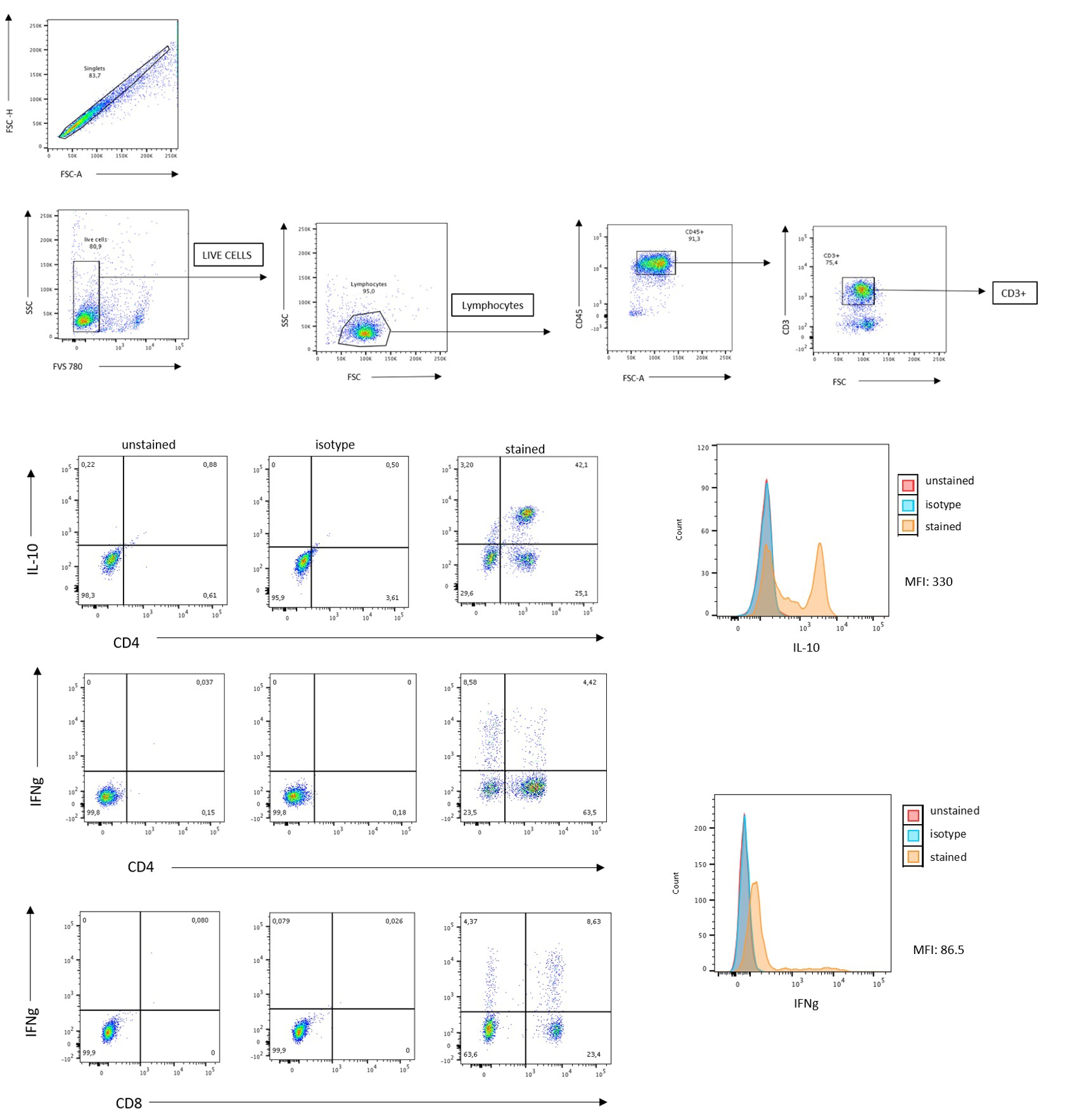

Supplement: Supplementary file 1 — Supplementary file1 [file 41598_2020_72711_MOESM1_ESM.tif]

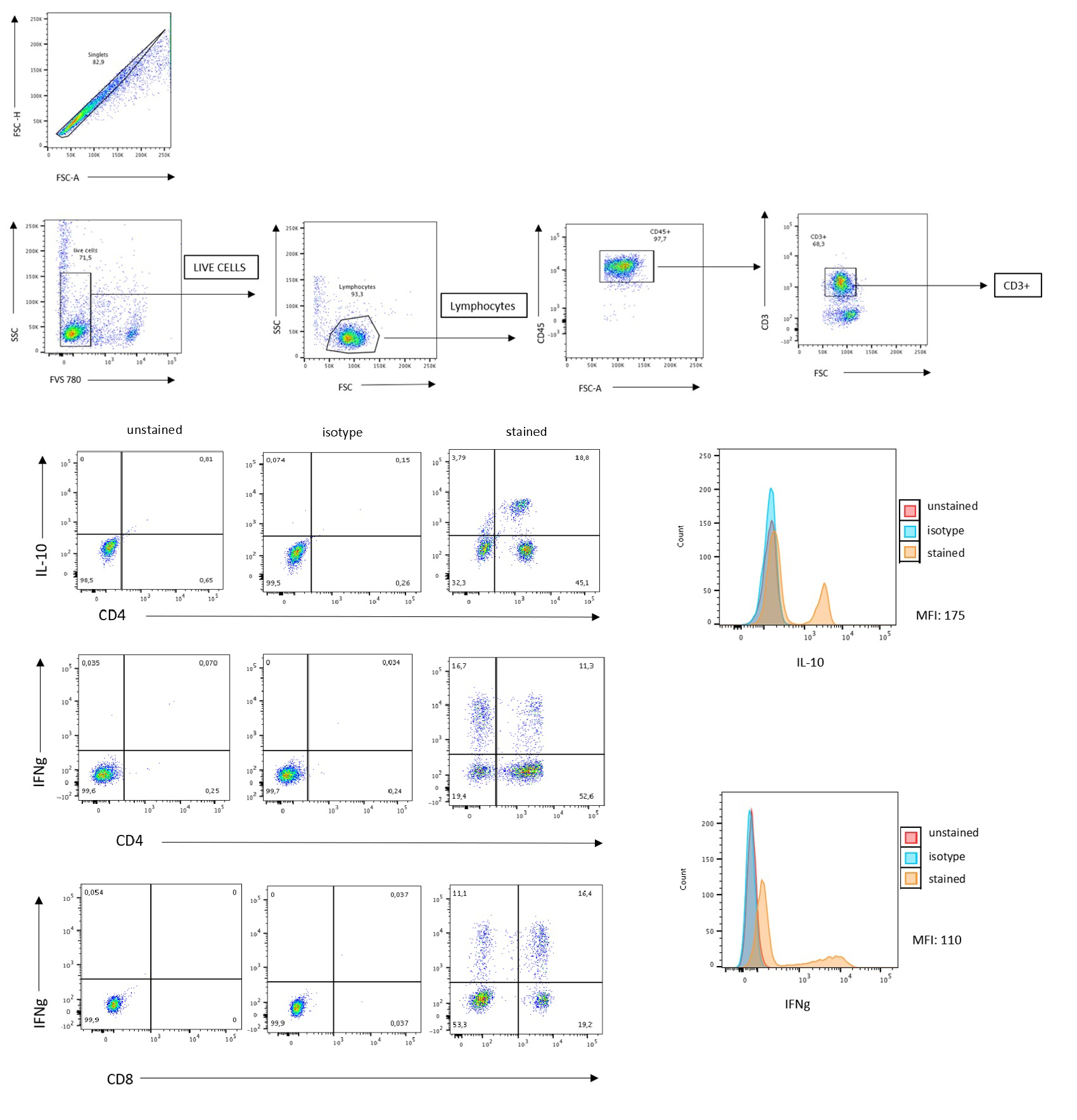

Supplement: Supplementary file 2 — Supplementary file2 [file 41598_2020_72711_MOESM2_ESM.tif]
