# Supplementary material for: Baseline IFN-γ and IL-10 expression in PBMCs could predict response to PD-1 checkpoint inhibitors in advanced melanoma patients
Source: Sci Rep. 2020 Oct 19;10:17626. doi: 10.1038/s41598-020-72711-2 (PMC7573589; doi:10.1038/s41598-020-72711-2)
Supplement: Supplementary file 3 — Supplementary file3 [file 41598_2020_72711_MOESM3_ESM.docx]

Baseline IFN-γ and IL-10 expression in PBMCs could predict response to PD-1 checkpoint inhibitors in advanced melanoma patients.

Emilio Francesco Giunta1,†, Giusi Barra1,†, Vincenzo De Falco1, Giuseppe Argenziano2, Stefania Napolitano1, Pasquale Vitale1, Nicoletta Zanaletti1, Marinella Terminiello1, Erika Martinelli1, Floriana Morgillo1, Davide Ciardiello1, Raffaele De Palma3, Fortunato Ciardiello1, Teresa Troiani1,*

1 Department of Precision Medicine, University of Campania Luigi Vanvitelli, Naples, 80131, Italy

2 Department of Mental and Physical Health and Preventive Medicine, University of Campania Luigi Vanvitelli, Naples, 80131, Italy

3 Department of Internal Medicine, University of Genoa - IRCCS Ospedale Policlinico San Martino, Genoa, 16132, Italy

† these authors contributed equally

[*teresa.troiani@unicampania.it](mailto:*teresa.troiani@unicampania.it) (corresponding author)

Supplementary figure legends:

Supplementary Figure S1. Example of gating strategy relative to one of the "non-responder" patients who had surprisingly high IL-10 levels.

Supplementary Figure S2. Example of gating strategy relative to one of the "responder" patients.
